# Endobronchial localization of Hodgkin’s disease

**DOI:** 10.11604/pamj.2017.28.9.11315

**Published:** 2017-09-07

**Authors:** Lamyae Amro, Siham Jridi, Hafsa Sajiai, Hind Serhane, Salma Aït batahar

**Affiliations:** 1 Department of Pneumology, CHU Mohammed VI, Laboratory PCIM, FMPM, UCA, Marrakech, Morocco

**Keywords:** Hodgkin´s disease, endobronchial, bronchoscopy, diagnosis

## Abstract

The endobronchial localization of Hodgkin's disease is a rare entity which is often confused with endobronchial tuberculosis in our setting. We report the case of a 16 years old female who presented with 6 months history of dry cough, hemoptysis, dyspnea, dysphagia and dysphonia. The chest radiography showed a mediastinal and pulmonary opacity. The chest CT scan found enlarged mediastinal lymph nodes. The bronchial biopsy and peripheral lymph node biopsy confirmed Hodgkin's disease with endobronchial localization. The patient received chemotherapy (ABVD protocol) and radiotherapy with a favorable follow up.

## Introduction

Mediastinum is the most common localization of lymphomas [1, 2]. The endobronchial localization is rare and usually presents a difficulty of differential diagnosis with tuberculosis; especially in highly endemic countries such as Morocco [3]. We report a case which illustrates the clinical, imagery and endoscopic features of endobronchial Hodgkin's disease with a literature review.

## Patient and observation

**Figure 1:**
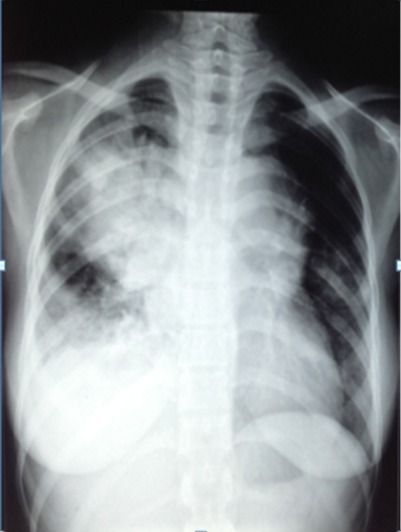
Anterior mediastinal-pulmonary opacity associated with a right pleural

**Figure 2:**
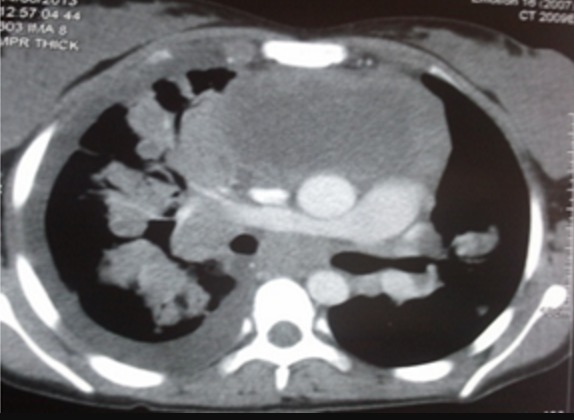
Complex anterior mediastinal lymph-tumor necrotic, alveolar condensations accented right and right pleural effusion

**Figure 3:**
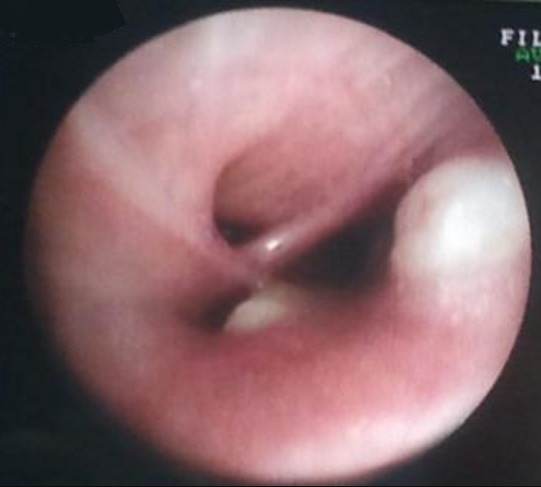
White granuloma at the entrance of the right upper lobe bronchus

**Figure 4:**
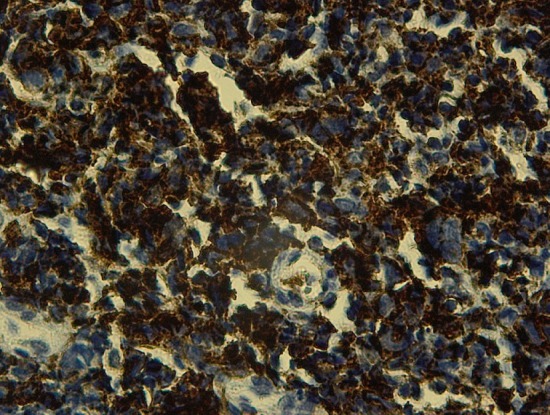
Immunohistochemistry; cytoplasmic expression with tumor cells périgolgien strengthening anti CD15 Ab (×40)

A 16 years old female without any prior medical history presented to our department for a six months history of productive cough; which was associated with minimal hemoptysis, anterior chest pain, dyspnea, dysphagia, dysphonia and a secondary amenorrhea. She had fatigue, weight loss and night sweats. The physical examination revealed absent tactile fremitus, dullness to percussion and decreased breath sounds in the lower part of the right side of the thorax. There were crackling rales in the upper part of the right side of the chest in addition to percussion dullness. Enlarged cervical lymph nodes were found; the largest one had a 2 cm diameter. Chest X ray showed a heterogeneous pulmonary and mediastinal opacity associated to a pleural effusion (Figure 1). The complete blood count showed a neutrophilic leukocytosis (2752/ml), thrombocytosis and hypochromic microcytic anemia (hemoglobin at 10 g/l). The C-reactive protein (CRP) was increased at 197mg/l, the sedimentation rate (sed-rate) was at 111, the Lactate Dehydrogenase test (LDH) was at 342 u/l and a hypocalcaemia. The liver and kidney functions tests were normal. The tuberculin skin test and the sputum acid fast bacilli were negative. The chest CT scan showed mediastinal lymphadenopathies, enlarged axillary lymph nodes in addition to pulmonary nodules and alveolar condensation (Figure 2). The abdominal CT scan showed mesenteric lymphadenopathies and a hepatomegaly. The cervical ultrasound found a thyroid nodule and bilateral necrotic cervical lymphadenopathies. The bronchoscopy showed four white lesions at the entrance of the middle lobe bronchus and the right upper lobe bronchus (Figure 3). A pathology study with immunohistochemistry of the biopsies was performed and concluded to an endobronchial localization of Hodgkin's lymphoma (Figure 4). The pathology of the pleural biopsy did not show any specific pattern. The pathology study of cervical lymph node biopsy showed histological patterns of Hodgkin´s disease as well as nodular sclerosis. The final diagnosis was stage IV Hodgkin's lymphoma (according to the Ann Arbor staging). A pre-treatment assessment was performed, it included HIV serology which was negative, ECG and echocardiogram which did not find any abnormalities, the spirometry was normal. Chemotherapy was indicated, the patient received ABVD protocol (Adriamycin, Bleomycin, Vinblastine, Dacarbazine) followed by radiotherapy. The clinical and radiological follow up was favorable.

## Discussion

Mediastinal involvement is the most common presentation of Hodgkin´s disease [3]. It represents 67% to 87% of cases and the main lesions are lymphadenopathies [4]. Endobronchial localization is a well-known presentation of lymphomas. It is often under diagnosed and its frequency is variable according to the authors. It might be more than 14% according to autopsy data [4, 5]. In Gallagher's series; only 3 patients had endobronchial localization of lymphoma which was confirmed by bronchial biopsy [4-6]. Another study included 469 lymphoma cases and among them, only 9 patients had associated endobronchial localization [3]. An Indian study reported a single case of endobronchial Hodgkin´s disease in a 12 year-old-girl, in this case; the lymphoma was revealed by a pneumonia that was rebellious to medical treatment [4]. The pathogenesis of tracheobronchial lesions during Hodgkin´s disease remains controversial [5], the hypothesis of hamatogenic dissemination from an occult extrathoracic focus is advanced. However, reaching the trachea or the bronchi by contiguity from a hilar or mediastinal lymphadenopathy seems to be the most convincing hypothesis [7]. Kiani et al [4-8] reported that the mean age was 42 years old and that male patients prevailed. Symptomatology was mainly made of cough in all patients, hemoptysis in half of the cases and dyspnea. Our patient presented with these same symptoms. On imagery, atelectasis is the most frequently found anomaly (in 2/3 of cases), in some cases, an isolated hilar mass was reported. The enlarged nodes are almost unfailing [7]. This was the case of our patient who had cervical, axillary and mediastinal lymphadenopathies. The endoscopy shows a budding lesion, which is usually very fragile and hemorrhagic. Visually, it cannot be distinguished from a primary lung cancer [7]. In the case at hand, the bronchoscopy showed friable granulations with necrotic spots. On respiratory function, an obstructive ventilatory disorder is typically observed. The treatment is based on chemotherapy and radiotherapy [4-8]. Radiation therapy is the treatment of choice in localized forms. The prognosis is determined by the early diagnosis and the stage of the disease.

## Conclusion

The endobronchial Hodgkin´s disease localization is rare, or is it underdiagnosed. A systematic exploration of the trachea and bronchi should be recommended for two reasons: on the one hand it allows the assessment of regional endobronchial extension and in the other hand, the biopsy of these lesions contributes to the histological diagnosis.

## Competing interests

The authors do not declare any conflict of interest.
